# Development of 2400–2450 MHz Frequency Band RF Energy Harvesting System for Low-Power Device Operation

**DOI:** 10.3390/s24102986

**Published:** 2024-05-08

**Authors:** Nasir Ullah Khan, Sana Ullah, Farid Ullah Khan, Arcangelo Merla

**Affiliations:** 1 Department of Engineering and Geology, Università degli Studi “G. d’Annunzio” Chieti—Pescara, 65127 Pescara, Italy; arcangelo.merla@unich.it; 2 Department of Electrical and Information Engineering, Politecnico di Bari, 70126 Bari, Italy; 3 Department of Mechatronics Engineering, University of Engineering and Technology, Peshawar 25000, Pakistan

**Keywords:** energy harvesting, impedance matching, rectifier circuit, radio frequency, patch antenna

## Abstract

Recently, there has been an increasing fascination for employing radio frequency (RF) energy harvesting techniques to energize various low-power devices by harnessing the ambient RF energy in the surroundings. This work outlines a novel advancement in RF energy harvesting (RFEH) technology, intending to power portable gadgets with minimal operating power demands. A high-gain receiver microstrip patch antenna was designed and tested to capture ambient RF residue, operating at 2450 MHz. Similarly, a two-stage Dickson voltage booster was developed and employed with the RFEH to transform the received RF signals into useful DC voltage signals. Additionally, an LC series circuit was utilized to ensure impedance matching between the antenna and rectifier, facilitating the extraction of maximum power from the developed prototype. The findings indicate that the developed rectifier attained a peak power conversion efficiency (PCE) of 64% when operating at an input power level of 0 dBm. During experimentation, the voltage booster demonstrated its capability to rectify a minimum input AC signal of only 50 mV, yielding a corresponding 180 mV output DC signal. Moreover, the maximum power of 4.60 µW was achieved when subjected to an input AC signal of 1500 mV with a load resistance of 470 kΩ. Finally, the devised RFEH was also tested in an open environment, receiving signals from Wi-Fi modems positioned at varying distances for evaluation.

## 1. Introduction

**Figure 1:**
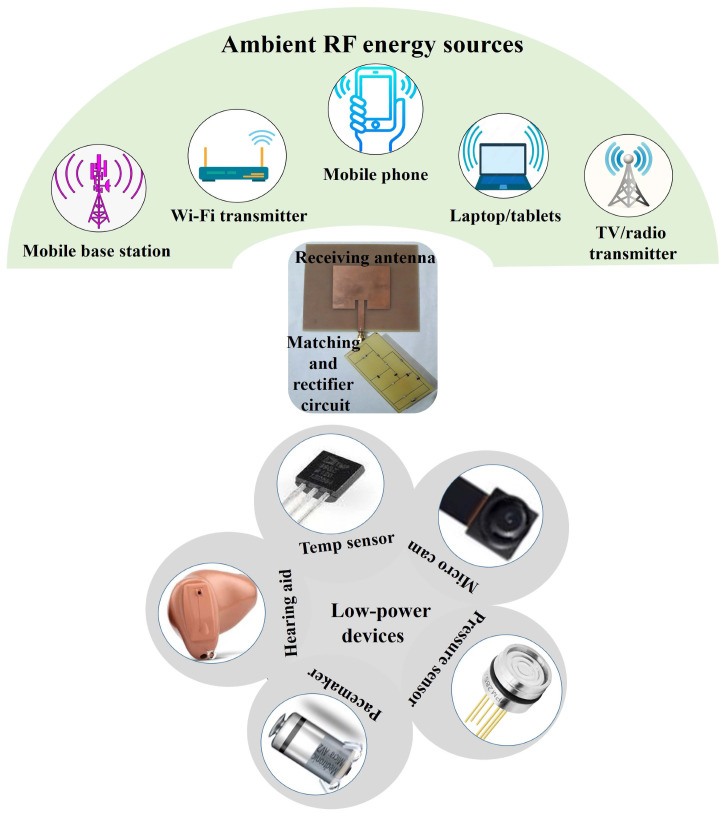
An overview of the RF energy harvester intended for energizing wireless sensors and portable gadgets using signals from multiple RF transmitters in the surroundings.

Over the past decade, energy sustainability has gained a notable preference to power portable and low-power appliances and gadgets. This shift is attributed to the advancements in nano and micro-electromechanical systems (NEMs and MEMs), microelectronics, ultra-large-scale integration (ULSI), and system-on-a-chip (SOC) technologies. Consequently, portable devices have assumed a pivotal role in crucial sectors, such as medical applications, security, communication, and industrial systems monitoring [1]. In situations where devices must be deployed in remote or difficult-to-access areas, making battery reinstatement and maintenance impractical, a successful approach involves the adoption of renewable energy harvesting [2,3]. This approach aims to eliminate the reliance on batteries or enhance the battery’s life cycle. Various energy sources, such as solar [4], wind [5], vibration [6], and acoustic energy [7], present in the environment have been efficiently converted into useful electrical energy to power low-power devices. Moreover, as a part of these energy sources, the extensive utilization of wireless communication has resulted in the pervasive presence of residual RF energy in the surroundings. The RF spectrum spans from very high frequencies (VHFs) to extremely high frequencies (EHFs), covering the range from 3 kHz to 300 GHz within the electromagnetic spectrum. All familiar transmission systems leverage specific segments of the RF spectrum to transmit signals to the receiving end [8]. RF energy is currently being broadcast by millions of transmitters globally. These transmitters consist of a wide range of devices, including mobile base stations, television and radio broadcasters, mobile phones, handheld radios, and Wi-Fi devices, as shown in Figure 1.

Table 1 displays the power consumption requirements for various low-power sensors and biomedical devices (shown in Figure 1). The data illustrate that a cardiac pacemaker can function within a low input power ranging from 1 to 10 µW, with an operational input voltage from 2 to 5.5 V. Similarly, a glucose sensor and a smartwatch can operate with 3 µW and 1 µW, respectively, requiring input voltages of 3.3 V and 1.5 V.

In scenarios where these devices need deployment in challenging or remote areas where battery maintenance and replacement are impractical, RF energy harvesting emerges as a successful approach. This method has the potential to either eliminate the need for a battery or extend the lifespan [15].

**Figure 2:**
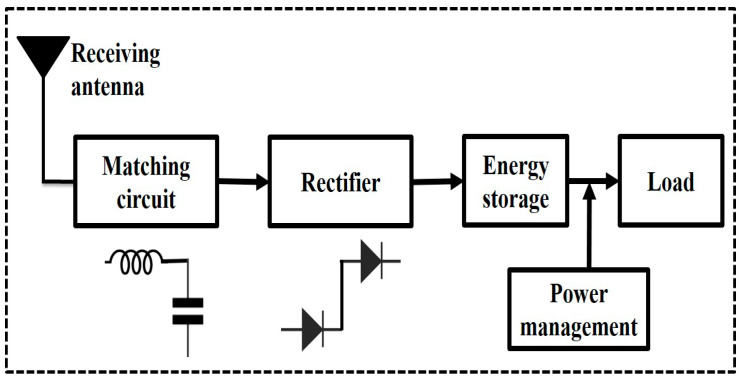
Schematic of the RF energy harvesting system.

RF energy harvesting is a method that entails the substantial conversion of residual RF signals present in the environment into useful electrical energy, facilitated by a combined receiving antenna and a rectifier, commonly referred to as a rectenna [16]. A standard RFEH setup includes a receiving antenna, impedance-matching circuit, rectifier, and energy storage component as shown in Figure 2.

The receiving antenna stands out as a crucial component in RFEH. Over the years, various geometric configurations of receiving antennas have been suggested in the literature to optimize harvesting efficiency. For example, Sunanda et al. [14] suggested a long periodic wideband antenna designed specifically for RFEH. The developed prototype demonstrated the capability to harvest 0.678 V from a −20 dBm input, achieving a maximum efficiency of 52%. Similarly, a circular patch antenna featuring two circular and rectangular slots was developed for RFEH [17]. This antenna presented an optimal choice for RFEH, boasting a wide bandwidth of 1590 MHz, a high gain of 2.81 dBi, and a compact size. Moreover, it is imperative to have an optimal matching network to maximize the power transfer to the rectifier circuit. Additionally, selecting an optimized matching network topology is crucial for minimizing insertion loss and maximizing power transfer efficiency. Various topologies, including L-section, T-section, or Pi-section, based on lumped electronic-circuit components, transmission lines, and stubs, present distinct trade-offs between losses and performance, necessitating careful consideration during the design process [18,19]. The rectifier holds significant importance in determining the overall efficiency of an RFEH system, given that the RF signals captured are initially in the form of narrowband AC voltage signals characterized by lower power density [20]. It is essential to rectify these signals to convert them into useful DC voltage signals [21]. The development of efficient rectifiers and voltage boosters has been a prominent focus of research over the years. For instance, Nastouh et al. [22] proposed a single-port voltage doubler specifically designed for harvesting GSM (global system for mobile communication) signals. The measured results indicated that the rectifier could attain a power conversion efficiency (PCE) of 26% for a −20 dBm input. Likewise, Surajo et al. developed a quad-band rectifier for the utilization of RFEH [23]. The DC voltage extracted from ambient RF signals was 0.433 V, with the rectifier achieving a maximum efficiency of 31.7% at 1.82 GHz across the quad-band when subjected to a −20 dBm input. Full-wave rectifiers, for example, the Dickson and cross-coupled differential-drive (CCDD) types, are frequently selected for RF energy harvesting due to their advantageous features, such as high voltage and PCE [24]. However, these rectifiers may face losses related to threshold voltage and leakage current, respectively. Although it is possible to minimize these losses, doing so often results in increased circuit complexity and overall size expansion. Therefore, selecting a particular rectifier type involves balancing various factors, including the nature of the application, available space, ambient input power, and desired output voltage.

This paper unveils a novel advancement in RF energy harvesting designed to charge portable gadgets with minimal operating power requirements. A high-gain (7.31 dBi) receiver microstrip patch antenna was designed and tested to capture ambient RF residue, operating at 2450 MHz. In parallel, a two-stage Cockcroft–Walton voltage booster was developed and integrated with the RFEH to convert and amplify the received signals into DC signals, which could be effectively utilized by low-power sensors and biomedical devices.

## 2. Modeling and Simulation

The design and simulation of the receiving antenna were facilitated using computer simulation technology (CST) 2019 software. Moreover, impedance matching and rectifier circuit design were accomplished through the utilization of Path Wave advanced design system (ADS) 2022 software.

### 2.1. Receiving Anntenna

**Figure 3:**
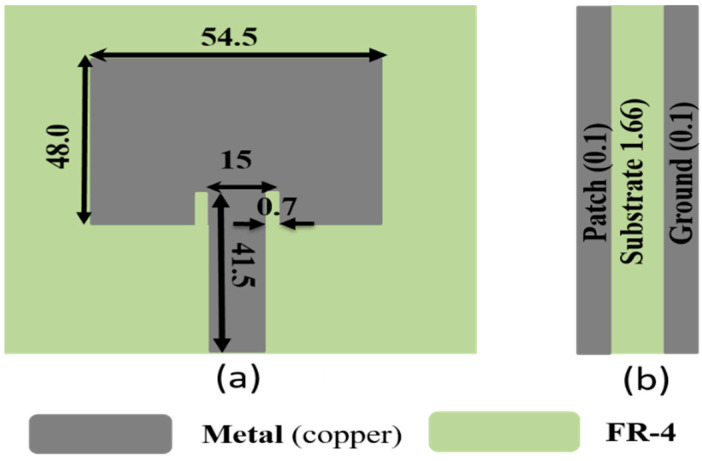
Proposed microstrip patch antenna: (**a**) front view; (**b**) side view. Unit: mm.

The microstrip patch antenna, as shown in Figure 3, is more suitable for RFEH due to its favorable characteristics, including its light weight, simple fabrication, broad bandwidth, and cost effectiveness. The antenna was designed using an FR-4 substrate having a dielectric constant ($\varepsilon_{\gamma })$ of 4.3 and a loss tangent (tan δ) of 0.025. The antenna comprised a rectangular patch, substrate, and a ground plane. The proposed antenna utilized an inset for impedance matching, as shown in Figure 3. The initial dimensions, width, and length of the patch can be computed by employing Equations (1) and (2).

(1) $$W=\frac{c}{2f\sqrt{\frac{\epsilon_{\gamma }+1}{2}}}$$

where *W* is the width of the patch, *f* is the resonating frequency, *c* is the speed of light, and $\varepsilon_{\gamma }$ represents the dielectric constant of the substrate.

(2) $$L=\frac{c}{2f\sqrt{\varepsilon_{ef}}}-0.824h\left(\frac{\left(\varepsilon_{ef}+0.3\right)\left(\frac{W}{h}+0.264\right)}{\left(\varepsilon_{ef}-0.258\right)\left(\frac{W}{h}+0.8\right)}\right)$$

where *L* is the length of the patch, *f* is the operating frequency, *c* represents the speed of light, *W* is the width of the patch, *h* is the height of the substrate, and $\varepsilon_{ef}$ represents effective dielectric constant, which can be computed using Equation (3).

(3) $$\varepsilon_{ef}=\frac{\varepsilon_{\gamma }+1}{2}+\frac{\varepsilon_{\gamma }-1}{2}\left[\frac{1}{\sqrt{1+12\left(\frac{h}{W}\right)}}\right]$$

The design of the inset aimed to establish the input impedance when looking at the edge of the patch to a predetermined target impedance. This was facilitated by exploiting the co-planarity between the antenna and the feedline, generating capacitance along the input of the feedline. The goal was to achieve a target input impedance equivalent to the feedline’s impedance connected to the patch antenna, typically set at 50 Ω. The depth of the inset could be computed using Equation (4).

(4) $$D=\frac{L}{\pi }{cos}^{-1}\left(\sqrt[{4}]{\frac{Z_{feedline}}{Z_{antenna}}}\right)$$

where *L* represents the length of the patch, $Z_{feedline}$ is the feedline’s impedance, and $Z_{antenna}$ is the antenna’s impedance.

### 2.2. Matching and Rectifier Circuit Design

**Figure 4:**
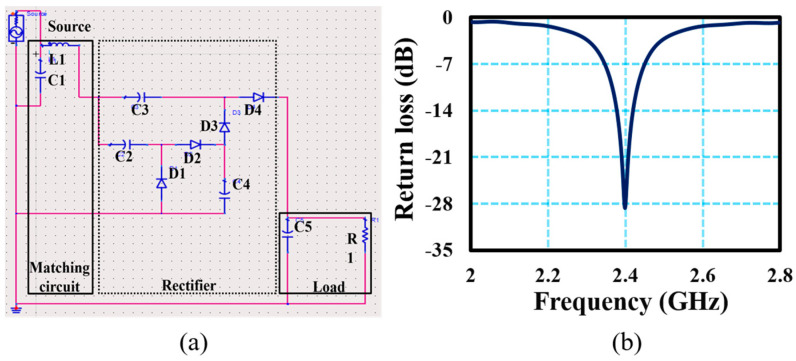
Dickson voltage booster for RFEH: (**a**) Designed impedance-matching and rectifier circuit; (**b**) simulated reflection coefficient of the rectifier.

A two-stage Dickson voltage booster was designed to transform the received RF signals into DC signals. The rectifier included four diodes (D1, D2, D3, and D4), four capacitors (C2, C3, C4, and C5), and a load resistor RL, as shown in Figure 4a. The rectifier circuit was developed to minimize reflections and achieve maximum PCE. To ensure optimal power transfer to the rectifier, it is crucial to align the impedance of the antenna to that of the rectifier, which can be calculated using the return loss Equation (5).

(5) $${Г=S}_{11}=\frac{Z_{REC}\;-{\;Z}_{ANT}}{Z_{REC}\;+{\;Z}_{ANT}}$$

where *Z_REC_* represents the rectifier’s impedance while *Z_ANT_* is the antenna’s impedance. The impedance matching was accomplished through a series combination of the inductor L1 (14 nH) and capacitor C1 (2 pF), as depicted in Figure 4b. Due to the low harvested voltage level, it was crucial to amplify it for effective use in low-power devices. The output voltage of a single-stage rectifier could be computed using Equation (6).

(6) $$V_{out1}=2V_{p}-V_{t1}-V_{t2}$$

where $V_{p}$, $V_{t1}$, and $V_{t2}$ represent the peak input voltage and threshold voltage levels of the first and second diode/transistor, respectively. Likewise, for a rectifier with multiple stages (*n*), the output voltage could be computed as the product of $V_{out1}$ and n, as shown in Equation (7).

(7) $$V_{out,n}=nV_{out1}$$

**Figure 5:**
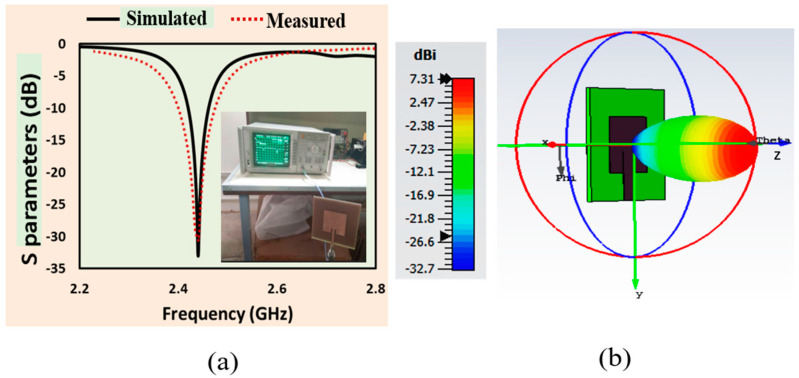
Proposed antenna analysis: (**a**) simulated and measured reflection coefficient (S11) of the designed antenna; (**b**) gain and 3D radiation pattern of the designed antenna.

The reflection coefficient (S11), radiation pattern, gain, output voltage, and PCE of the proposed antenna and rectifier were simulated. Figure 5a displays the simulated and measured reflection coefficients of the proposed antenna, while Figure 5b illustrates the gain and 3D radiation pattern of the designed antenna. The S parameters of the designed antenna were obtained through measurements with the help of a vector network analyzer (VNA). The simulated and measured results demonstrate strong agreement, indicating that the designed antenna resonated with a −10 dB measured bandwidth of 75 MHz. The radiation characteristics of the antenna were assessed through the analysis of its far-field radiation pattern and gain at the corresponding operational frequency of 2.45 GHz. At the resonance frequency, the antenna achieved a high gain of 7.31 dBi, making it a suitable choice for RFEH applications.

**Figure 6:**
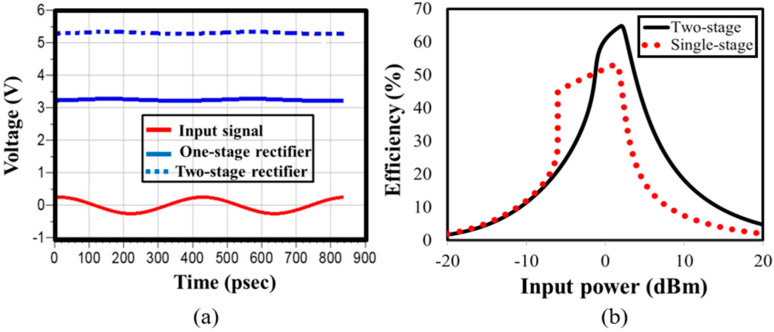
Developed rectifier circuit: (**a**) simulated input and output voltage signals of the rectifier; (**b**) simulated PCE of the proposed rectifier with 0 dBm input power.

The proposed rectifier circuit underwent simulation, considering the input and output voltage signals. The input voltage signal was simulated at 0 dBm, having a frequency of 2.45 GHz and an impedance of 50 Ω, aligning with the parameters of the receiving antenna. Figure 6a showcases the simulated output voltage achieved using the developed two-stage rectifier. The simulated results demonstrate the achievement of a DC output voltage of 5.2 V at 0 dBm input power, showcasing suitability for operating portable gadgets and other low-power appliances. Figure 6a further provides a comparison with a single-stage rectifier, displaying an output voltage of 3.2 V. Utilizing two stages for the same input signal could enhance the output voltage to 5.2 V.

In the field of energy harvesting, the PCE of the rectifier holds significant importance, representing how efficiently the rectifier can convert input signals into practical DC voltage. The PCE is quantified through Equation (7).

(8) $$\eta \left(\%\right)=\frac{P_{out}}{P_{in}}\times 100\%=\frac{I_{DC}\times V_{DC}}{P_{in}}\times 100\%$$

Figure 6b illustrates the efficiency of the developed rectifier circuit, displaying advancements over prior versions and achieving an impressive 64% efficiency. The efficiency simulation of the designed rectifier aligned with the parameters of the receiving antenna, including a 50 Ω impedance, 2.45 GHz frequency, and 0 dBm input power equivalent to the ambient input power from Wi-Fi modems. Figure 6 also features a comparative analysis, showcasing the efficiency of the designed rectifier compared to a single-stage rectifier. With a single-stage rectifier’s utilization, a maximum efficiency of 53% was achieved, which was enhanced up to 64% with the developed two-stage rectifier, achieved through proper impedance matching under identical input parameters.

Figure 6b illustrates the efficiency of the developed rectifier circuit, displaying advancements over prior versions and achieving an impressive 64% efficiency. The efficiency simulation of the designed rectifier aligned with the parameters of the receiving antenna, including a 50 Ω impedance, 2.45 GHz frequency, and 0 dBm input power. The Figure 6b also features a comparative analysis, showcasing the efficiency of the designed rectifier compared to a single-stage rectifier. With a single-stage rectifier’s utilization, a maximum efficiency of 53% was achieved, which was enhanced up to 64% with the developed two-stage rectifier, achieved through proper impedance matching under identical input parameters.

### 2.3. Fabrication

**Figure 7:**
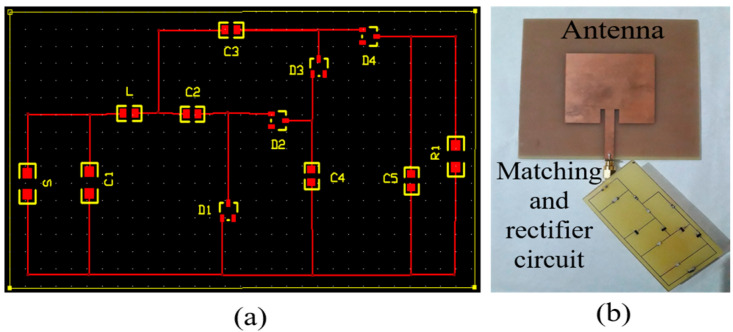
Prototype device: (**a**) PCB layout of the rectifier circuit; (**b**) fabricated RFEH system.

The fabrication of the designed antenna’s PCB was completed, comprising a copper patch and ground plane on an FR-4 sheet ${(\varepsilon }_{\gamma }=4.3$ and tan δ = 0.025), having a thickness of 1.66 mm. The circuit layout was generated using Express PCB, as shown in Figure 7a. The matching and rectifier circuit were implemented on a wafer with dimensions of 76.2 × 38.1 mm^2^. Considering the circuit’s low voltage and low input power characteristics, commercially available Schottky diodes HSMS-2850 (from Avago) were selected for rectification due to their advantageous features, such as a low voltage drop, low junction capacitance, and high switching speed, leading to enhanced efficiency at higher frequencies. Additionally, surface-mount capacitors were utilized and assumed to be connected with the diodes.

In the layout design phase, track pads for the diodes were initially designed, with an area of 0.98 mm^2^ allocated for each pad where the diodes were to be mounted. Similarly, for the matching circuit, track pads for the inductor and capacitor of size SMT-1206 (from Avago) were generated, each having an area of 1.77 mm^2^. The rectifier circuit, comprising the diodes and four capacitors, employed track pads sized SMT-0805 (from Avago) with an area of 1.01 mm^2^. All track pads for the inductors, capacitors, and diodes were interconnected through trace lines. After the layout design, the PCB was developed, and all components were mounted onto it. An SMT Rework station machine, operating at a high temperature (180 °C) was used to solder the diodes and SMT capacitors onto the developed PCB board. An SMA connector (from Avago) facilitated the connection between the antenna and the rectifier circuit. Figure 7b illustrates the completely developed RFEH prototype.

## 3. Results and Discussion

**Figure 8:**
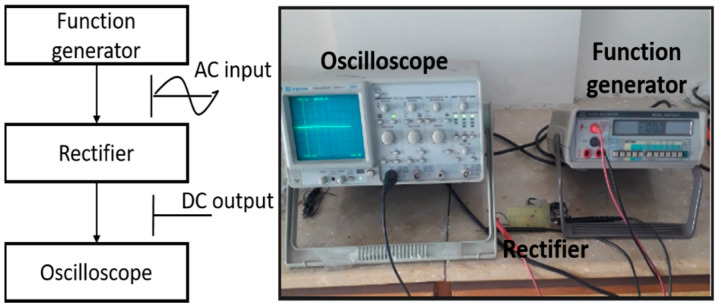
Experimental arrangement for evaluating the developed rectifier.

Figure 8 presents the experimental setup employed in the laboratory for the testing of the rectifier’s prototype. During the experimental phase, the rectifier was interfaced with a function generator to generate diverse input AC voltage signals with varying amplitudes and frequencies. Simultaneously, an oscilloscope was employed to connect with the rectifier, enabling the assessment and observation of multiple parameters of the input and output voltage signals, including frequency and amplitude.

**Figure 9:**
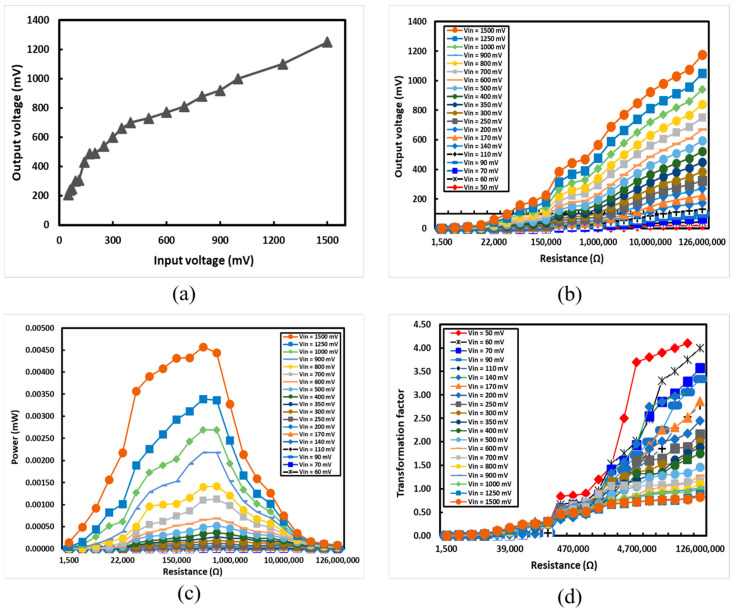
Testing of the developed rectifier: (**a**) the output voltage plotted against the input voltage for the developed rectifier; (**b**) the output voltage plotted against different load resistors linked to the rectifier; (**c**) the output power plotted against different load resistors linked to the rectifier; (**d**) the transformation factor against different load resistors for the developed rectifier.

The developed rectifier underwent testing with various input voltage levels, spanning from 50 mV to 1.5 V. Figure 9a illustrates the output voltage signals plotted against various input voltage levels. The developed rectifier demonstrated its capability to rectify both lower voltage levels (in mV ranges) and higher voltage levels. The rectifier effectively converted a minimum input voltage of 50 mV AC to 180 mV DC, showcasing its efficiency in voltage amplification. The rectifier exhibited a more pronounced amplification response for low voltage levels, aligning with the intended objective, given that ambient RF signals typically carry low voltage levels. Similarly, the rectifier efficiently rectified a maximum input voltage of 1.5 V.

The response of the developed rectifier was evaluated under different load resistors. A range of load resistors, with values spanning from 1.5 kΩ to 126 MΩ, were connected to the rectifier, and the output DC voltage was scaled across each resistor in the experimental phase. Various input signals, varying from 50 mV to 1.5 V, were applied across different resistance values, and the resulting output voltage is depicted in Figure 9b.

The power transferred to the load resistance (RL) could be evaluated by examining the output DC voltage (VDC). Figure 9c displays a graph depicting the relationship between the output power and load resistance. The experimental setup incorporated various input AC voltage levels, ranging from 50 mV to 1.5 V. Measurements of output power were conducted across a range of resistors. Irrespective of the amplitude of the input AC voltage, the maximum load power was attained with a load resistor of 470,000 Ω. This observation implies that opting for a 470,000 Ω load resistance could yield optimal power delivery with the developed rectifier. The maximum power recorded was 4.59 × 10^−6^ W, attained with an input AC signal of 1.5 V, having the load resistance set at 470,000 Ω.

The transformation factor (T factor), represented by the ratio of output DC voltage to input AC voltage (Vout/Vin), is plotted against various load resistance (RL) levels in Figure 9d. To analyze the T factor with load resistance, a range of input AC voltage signals (from 50 mV to 1.5 V) was applied across diverse load resistances (ranging from 1.5 kΩ to 126 MΩ). The maximum T factor, reaching 4.10, was attained for an input AC voltage of 50 mV with a load resistance of 76,000 kΩ. Table 2 offers a comprehensive overview of the analysis conducted on the developed AC to DC rectifier prototype.

The power delivered to the load (*R_L_*) could be determined by measuring the output DC voltage (*V_DC_*) across the load resistance, as indicated in Equation (9).

(9) PDC=V2DCRL

where $V_{DC}$ is the rectified voltage while $R_{L}$ is the load resistance.

**Figure 10:**
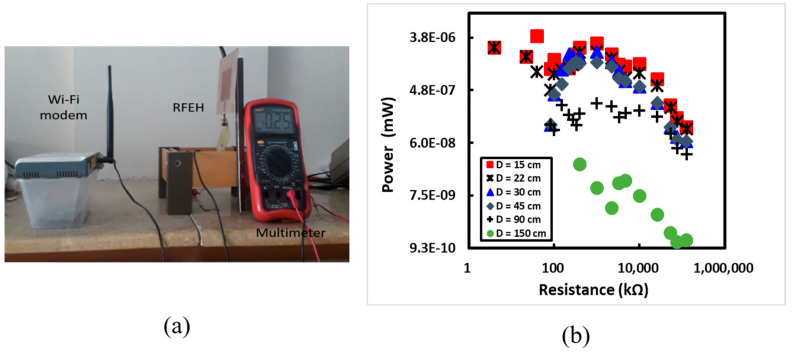
Testing of the developed RFEH system in a real environment: (**a**) the experimental arrangement for evaluating the developed RFEH; (**b**) the harvested power against different load resistors for the RFEH system.

Figure 10a illustrates the experimental setup for real environment measurements, where the developed RFEH system is tested to receive RF signals from a WiFi modem acting as a source. The experimental configuration incorporated a Wi-Fi modem as the source of the RF signals. Measurements were conducted at distances ranging from 15 cm to 150 cm from the source. Figure 10b demonstrates that the maximum output power of 0.47 nW was achieved when positioned 15 cm away from the source, indicating that signals with high power density can be captured using the receiving antenna in close proximity to the source. Nevertheless, as the separation between the source and receiving antenna increased, the received power diminished, following an inverse square relationship. It is noteworthy that the harvested power was relatively low, attributed to the fact that a Wi-Fi modem transmits RF signals with low transmitted power, typically in the range of a few µW.

Table 3 outlines a comparison between the developed RFEH system and previous studies within the RFEH field. The evaluation focuses on key characteristics, including the size, resonance frequency, output voltage and power, rectifier type, and PCE.

The GSM 900/1800 and ISM/Wi-Fi 2400 bands transmit a significant amount of RF signals in the surroundings and emerge as the predominant frequencies for RFEH [31]. In the literature, the majority of RFEH has been designed to harness RF energy residues from these bands, as they offer the highest power density in the surroundings, ranging from 36 nW/cm^2^ to 84 nW/cm^2^ [32]. Over the years, RFEH has undergone continuous development, incorporating various receiving antennas, diverse impedance-matching circuits, and a broad range of rectifier topologies. Among the different antenna geometries, patch antennas stand out as a favorable option due to their characteristics, including ease of fabrication, lightweight design, broad bandwidth, and cost effectiveness [33]. Designing an impedance-matching network necessitates a careful balance of factors, such as device dimensions, frequency, and adjustability. The choice between the transmission line and LC-based impedance matching depends on the device size and application type. However, Mutee et al. [34] revealed, in measured results, that below 2600 MHz, both matching topologies exhibit similar behavior. Similarly, incorporating multiple stages in rectifiers can be employed to enhance the output voltage level for a specific range of applications. However, this enhancement is accompanied by larger size and diminished efficiency [35,36].

Recent research has predominantly focused on improving the PCE of rectifiers in RFEH systems [37]. This emphasis arises due to the relatively low ambient power density, necessitating highly efficient rectifiers to effectively convert available power into usable electrical energy. For instance, a three-stage Dickson rectifier proposed for RFEH operating at 915 MHz achieved a modest PCE of 25% despite efforts to boost the output voltage to 4 V [25]. Similarly, a seven-stage full-wave rectifier intended for RFEH applications at 2400 MHz exhibited a measured PCE of 18.6%, coupled with a maximum output voltage of 2 V [20]. Xiaoqiang et al. [8] successfully increased the PCE to 52.5% by employing a multi-stage full-wave rectifier, yielding a maximum output voltage of 4.8 V while operating at a resonance frequency of 2437 MHz. In this work, the PCE was notably improved by implementing appropriate LC-based impedance matching and employing a two-stage Cockcroft rectifier. This enhanced configuration achieved a remarkable PCE of 64%, which presents an optimal solution for effectively utilizing the harvested voltage to power low-power devices.

## 4. Conclusions

In this paper, we developed a novel RF energy harvesting (RFEH) system, incorporating a microstrip patch antenna designed for the 2400–2450 MHz frequency band, an LC impedance-matching network, and an efficient two-stage voltage doubler for low-power device operation. The RFEH was fabricated on an FR-4 substrate through PCB fabrication. The system’s performance was thoroughly assessed using simulations and experimental tests. Our analysis demonstrated consistent and stable energy harvesting performance, featuring a high-gain antenna at 7.31 dBi, which exhibited stability and resonance, along with a notable impedance bandwidth within the specified frequency range.

The developed rectifier circuit showcased a robust power conversion efficiency of 64% at 0 dBm, yielding an output voltage of 1.3 V across various load resistances ranging from 1.5 kΩ to 126 MΩ. The rectifier’s capacity to boost low voltage levels was evident, exemplified by the successful rectification of a minimum input AC signal of 50 mV into a DC voltage of 180 mV. Moreover, with a load resistance optimized at 470 kΩ, the system achieved a power output of 4.60 × 10^−6^ W when supplied with an input AC signal of 1.5 V.

The obtained results and the successful demonstration underscore the significant potential of the developed RFEH system for powering low-power sensors and appliances. In our future work, we plan to develop an RFEH system with a multi-band receiving antenna to capture signals from diverse sources simultaneously. This will necessitate the implementation of a complex impedance network and rectifier circuit for different frequency bands. The focus will be on reducing the overall system size and optimizing the PCE of the rectifier, intended for its applications for various biomedical sensors.

## Figures and Tables

**Table 1:** Operating power demands of well-known low-power sensors and devices.

Low-Power Device	Temperature Sensor	Smoke Sensor	Glucose Sensor	Pacemaker	Cochlear Implant	Smartwatch
Reference	[9]	[10]	[11]	[12]	[13]	[14]
Voltage required (V)	2–3.3	3.3–12	3.3–5.5	2–5.5	1.5–5.5	1.5–3.3
Power required (µW)	400	1000	3	1–10	100–10,000	1
Power level	Low	Low	Extremely low	Extremely low	Low	Extremely low

**Table 2:** Overview of the experimental characterization of the developed rectifier.

Parameter	Value or Range
Minimum AC voltage rectified	50–180 mV
Maximum voltage rectified	1500–1300 mV
Input AC voltage levels	50–1500 mV
Load resistances	1.5–126,000 kΩ
Optimum load	470,000 Ω
Maximum T factor	4.10

**Table 3:** Comparison of the developed RF energy harvesting system with state-of-the-art technology.

References	[25]	[26]	[27]	[8]	[28]	[29]	[30]	This Work
Antenna size/ Rectifier size (mm × mm)	28.5 × 28/NA	27.5 × 25.90/14 × 27.5	89 × 89/55 × 20	53 × 43.4/13.15 × 8.23	53 × 43.5/22.07 × 26.2	NA	110 × 60/70 × 50	54.5 × 48/76.2 × 38.1
Resonance frequency (MHz)	2400	2400	915	2437	2452	915	915	2450
Matching network	Open-circuit stub	Transmission line stubs	N/A	Transmission line stubs	Transmission line stubs	LC-based	Short circuit stub	LC-based
Rectifier stages	Seven	Two	Five	Multiple	Single	Three	Single	Two
Substrate	FR-4	Rogers TMM10	Polymer	PTFE	PTFE + FR-4	CMOS	FR-4	FR-4
Output voltage (V)	2	2.6	2.9	4.8	2.8	4	2.3	5.2
PCE (%) @ input power (dBm)	18.6 @ −50	47.7 @ 11	20 @ 0	52.5 @ 7	33.8 @ 5	25 @ 1	25 @ 0	64 @ 0

## Data Availability

Data are contained within the article.

## References

[1] Basu A., Basu A.K., Ghosh S., Bhattacharya S. (2023). Introduction to MEMS Applications in Electronics and Engineering. MEMS Applications in Electronics and Engineering.

[2] Shi Y., Cui X., Qi L., Zhang X., Li X., Shen H. (2023). A Novel Energy Harvesting Method for Online Monitoring Sensors in HVdc Overhead Line. IEEE Trans. Ind. Electron..

[3] Piyarathna I.E., Lim Y.Y., Edla M., Thabet A.M., Ucgul M., Lemckert C. (2023). Enhancing the Bandwidth and Energy Production of Piezoelectric Energy Harvester Using Novel Multimode Bent Branched Beam Design for Human Motion Application. Sensors.

[4] Sarang S., Stojanović G.M., Drieberg M., Stankovski S., Bingi K., Jeoti V. (2023). Machine Learning Prediction Based Adaptive Duty Cycle MAC Protocol for Solar Energy Harvesting Wireless Sensor Networks. IEEE Access.

[5] Calautit K., Johnstone C. (2023). State-of-the-Art Review of Micro to Small-Scale Wind Energy Harvesting Technologies for Building Integration. Energy Convers. Manag. X.

[6] Zhang M., Song R., Zhang J., Zhou C., Peng G., Tian H., Wu T., Li Y. (2023). Research on Electromagnetic Vibration Energy Harvester for Cloud-Edge-End Collaborative Architecture in Power Grid. J. Cloud Comput..

[7] Li B., Chen H., Xia B., Yao L. (2023). Acoustic Energy Harvesting Based on Topological States of Multi-Resonant Phononic Crystals. Appl. Energy.

[8] Liu X., Li M., Chen X., Zhao Y., Xiao L., Zhang Y. (2023). A Compact RF Energy Harvesting Wireless Sensor Node with an Energy Intensity Adaptive Management Algorithm. Sensors.

[9] Alippi C., Anastasi G., Di Francesco M., Roveri M. (2009). Energy Management in Wireless Sensor Networks with Energy-Hungry Sensors. IEEE Instrum. Meas. Mag..

[10] Todaro M.T., Guido F., Algieri L., Mastronardi V.M., Desmaele D., Epifani G., De Vittorio M. (2018). Biocompatible, Flexible, and Compliant Energy Harvesters Based on Piezoelectric Thin Films. IEEE Trans. Nanotechnol..

[11] Liao Y.-T., Yao H., Lingley A., Parviz B., Otis B.P. (2012). A 3-ΜW CMOS Glucose Sensor for Wireless Contact-Lens Tear Glucose Monitoring. IEEE J. Solid-State Circuits.

[12] Tsui C.-Y. (2013). Energy Harvesting and Power Delivery for Implantable Medical Devices. Found. Trends Electron. Des. Autom..

[13] Yip M., Jin R., Nakajima H.H., Stankovic K.M., Chandrakasan A.P. (2015). A Fully-Implantable Cochlear Implant SoC with Piezoelectric Middle-Ear Sensor and Arbitrary Waveform Neural Stimulation. IEEE J. Solid-State Circuits.

[14] Roy S., Tiang J.-J., Bin Roslee M., Ahmed M.T., Kouzani A.Z., Mahmud M.A.P. (2022). Design of a Highly Efficient Wideband Multi-Frequency Ambient RF Energy Harvester. Sensors.

[15] Zada M., Iman U.R., Basir A., Yoo H. (2023). Battery-Free Digitally Embroidered Smart Textile Energy Harvester for Wearable Healthcare IoTs. IEEE Trans. Ind. Electron..

[16] Khan N.U., Khan F.U. RF Energy Harvesting for Portable Biomedical Devices. Proceedings of the 2019 22nd International Multitopic Conference (INMIC).

[17] Muhammad S., Smida A., Waly M.I., Mallat N.K., Iqbal A., Reza Khan S., Alibakhshikenari M. (2022). Design of Wideband Circular-Slot Antenna for Harvesting RF Energy. Int. J. Antennas Propag..

[18] Thompson M., Fidler J.K. (2004). Determination of the Impedance Matching Domain of Impedance Matching Networks. IEEE Trans. Circuits Syst. I Regul. Pap..

[19] Alibakhshikenari M., Virdee B.S., Shukla P., See C.H., Abd-Alhameed R.A., Falcone F., Limiti E. (2020). Improved Adaptive Impedance Matching for RF Front-End Systems of Wireless Transceivers. Sci. Rep..

[20] Cansiz M., Altinel D., Kurt G.K. (2019). Efficiency in RF Energy Harvesting Systems: A Comprehensive Review. Energy.

[21] Khan N.U., Khan F.U., Farina M., Merla A. (2024). RF Energy Harvesters for Wireless Sensors, State of the Art, Future Prospects and Challenges: A Review. Phys. Eng. Sci. Med..

[22] Nikkhah N., Keshavarz R., Abolhasan M., Lipman J., Shariati N. Efficient Dual-Band Single-Port Rectifier for RF Energy Harvesting at FM and GSM Bands. Proceedings of the 2022 Wireless Power Week (WPW).

[23] Muhammad S., Tiang J.J., Wong S.K., Smida A., Waly M.I., Iqbal A. (2021). Efficient Quad-Band RF Energy Harvesting Rectifier for Wireless Power Communications. AEU Int. J. Electron. Commun..

[24] Chun A.C.C., Ramiah H., Mekhilef S. (2022). Wide Power Dynamic Range CMOS RF-DC Rectifier for RF Energy Harvesting System: A Review. IEEE Access.

[25] Kadir E.A., Hu A.P., Biglari-Abhari M., Aw K.C. Indoor WiFi Energy Harvester with Multiple Antenna for Low-Power Wireless Applications. Proceedings of the 2014 IEEE 23rd International Symposium on Industrial Electronics (ISIE).

[26] DeLong B.J., Kiourti A., Volakis J.L. (2018). A Radiating Near-Field Patch Rectenna for Wireless Power Transfer to Medical Implants at 2.4 GHz. IEEE J. Electromagn. RF Microw. Med. Biol..

[27] Kim S., Bito J., Jeong S., Georgiadis A., Tentzeris M.M. A Flexible Hybrid Printed RF Energy Harvester Utilizing Catalyst-Based Copper Printing Technologies for Far-Field RF Energy Harvesting Applications. Proceedings of the 2015 IEEE MTT-S International Microwave Symposium.

[28] Liu X., Li M., Chen X., Zhao Y., Xiao L., Zhang Y. (2023). A Compact Stacked RF Energy Harvester with Multi-Condition Adaptive Energy Management Circuits. Micromachines.

[29] Li P., Long Z., Yang Z. (2021). RF Energy Harvesting for Batteryless and Maintenance-Free Condition Monitoring of Railway Tracks. IEEE Internet Things J..

[30] Loubet G., Takacs A., Dragomirescu D. (2019). Implementation of a Battery-Free Wireless Sensor for Cyber-Physical Systems Dedicated to Structural Health Monitoring Applications. IEEE Access.

[31] Divakaran S.K., Das Krishna D. (2019). Nasimuddin RF Energy Harvesting Systems: An Overview and Design Issues. Int. J. RF Microw. Comput. Eng..

[32] Muhammad S., Tiang J.J., Wong S.K., Smida A., Ghayoula R., Iqbal A. (2021). A Dual-Band Ambient Energy Harvesting Rectenna Design for Wireless Power Communications. IEEE Access.

[33] Christina G. (2021). A Review on Microstrip Patch Antenna Performance Improvement Techniques on Various Applications. J. Trends Comput. Sci. Smart Technol..

[34] ur Rehman M., Ahmad W., Khan W.T. Highly Efficient Dual Band 2.45/5.85 GHz Rectifier for RF Energy Harvesting Applications in ISM Band. Proceedings of the 2017 IEEE Asia Pacific Microwave Conference (APMC).

[35] Park S., Yang J., Rivas-Davila J. (2020). A Hybrid Cockcroft–Walton/Dickson Multiplier for High Voltage Generation. IEEE Trans. Power Electron..

[36] Chong G., Ramiah H., Yin J., Rajendran J., Wong W.R., Mak P.-I., Martins R.P. (2018). Ambient RF Energy Harvesting System: A Review on Integrated Circuit Design. Analog Integr. Circuits Signal Process..

[37] Chen D., Li R., Xu J., Li D., Fei C., Yang Y. (2023). Recent Progress and Development of Radio Frequency Energy Harvesting Devices and Circuits. Nano Energy.

